# SET: a pupil detection method using sinusoidal approximation

**DOI:** 10.3389/fneng.2015.00004

**Published:** 2015-04-09

**Authors:** Amir-Homayoun Javadi, Zahra Hakimi, Morteza Barati, Vincent Walsh, Lili Tcheang

**Affiliations:** ^1^Department of Experimental Psychology, Institute of Behavioural Neuroscience University College LondonLondon, UK; ^2^Young Researchers and Elite Club, Qazvin Branch, Islamic Azad UniversityQazvin, Iran; ^3^Institute of Cognitive Neuroscience, University College LondonLondon, UK

**Keywords:** eye tracking, pupil detection, dark pupil, head mounted device, ellipse fitting

## Abstract

Mobile eye-tracking in external environments remains challenging, despite recent advances in eye-tracking software and hardware engineering. Many current methods fail to deal with the vast range of outdoor lighting conditions and the speed at which these can change. This confines experiments to artificial environments where conditions must be tightly controlled. Additionally, the emergence of low-cost eye tracking devices calls for the development of analysis tools that enable non-technical researchers to process the output of their images. We have developed a fast and accurate method (known as “SET”) that is suitable even for natural environments with uncontrolled, dynamic and even extreme lighting conditions. We compared the performance of SET with that of two open-source alternatives by processing two collections of eye images: images of natural outdoor scenes with extreme lighting variations (“Natural”); and images of less challenging indoor scenes (“CASIA-Iris-Thousand”). We show that SET excelled in outdoor conditions and was faster, without significant loss of accuracy, indoors. SET offers a low cost eye-tracking solution, delivering high performance even in challenging outdoor environments. It is offered through an open-source MATLAB toolkit as well as a dynamic-link library (“DLL”), which can be imported into many programming languages including C# and Visual Basic in Windows OS (www.eyegoeyetracker.co.uk).

## Introduction

Eye-tracking is fundamental for accurate gaze-tracking. It is a powerful tool for the study of visual cognition and is used in both diagnostic and interactive applications (Duchowski, 2002, 2007). Employing eye-tracking in diagnostic applications, such as market research (Rayner et al., 2001; Müller et al., 2012) or the understanding of human attention in infants (Kato and Konishi, 2013) and adults (Kaspar et al., 2013), as well as cognitive disorders (von dem Hagen et al., 2013), provides an objective and quantitative measure of the viewer's point-of-regard (PoR) (for reviews see Goldberg and Wichansky, 2003; Hayhoe and Ballard, 2005). Interactive applications use viewers' PoR as a control input (Ward and MacKay, 2002; Oyekoya and Stentiford, 2006; Mele and Federici, 2012). Despite widespread application and significant progress, mobile eye-tracking, particularly in external environments, remains challenging due to factors such as occlusion and variability in scale and lighting (Hansen and Ji, 2010).

Early eye-trackers were static cameras placed on a table or attached to a monitor, far from the user (e.g., Tobii eye-trackers www.tobii.com and SMI eye-tracking glasses www.eyetracking-glasses.com). These setups have a software solution to detect the head and subsequently the eye(s) for further analysis. Placement of the camera on a pan-and-tilt device partially extends the camera's field of view (e.g., EyeFollower www.interactive-minds.com), yet the physical range of these systems is still limited (Ohno et al., 2002; Lemahieu and Wyns, 2011). Greater accuracy was achieved, at the expense of freedom of movement, by mounting the eye-tracker on a chin rest, upon which the participant's head is also fixed (e.g., EyeLink www.sr-research.com). These systems, requiring a fixed-head, restrict the participant's freedom of movement. It should be mentioned that these setups are used in many applications, such as when high tracking precision is required.

In order to have ultimate freedom of movement and portability, a second group of eye-trackers were developed where the camera is mounted directly onto the head of the user (“mobile eye-trackers”), allowing the user to be free to walk around (e.g., www.ergoneers.com, Tobii and SMI). Most of these systems can be used in both indoor and outdoor applications. Examples of the application of these systems include market research and developmental studies (Franchak et al., 2010; Graham et al., 2012; Oakes, 2012). As more portable external eye-trackers enter the market, a new challenge emerges where pupil detection must be maintained under uncontrolled and potentially wildly varying light conditions, as opposed to the static and controlled light conditions in a laboratory environment.

Considering the rapid advancement of the hardware, the challenging part of eye-tracking is now the software that processes the images acquired by the camera. Many low-cost image capture mechanisms have been proposed, mostly composed of off-the-shelf components (Li et al., 2006; Schumann et al., 2008; Lemahieu and Wyns, 2011; Huang et al., 2013). These methods either use new approaches (Mäenpää, 2005; Asadifard and Shanbezadeh, 2010; Lemahieu and Wyns, 2011) or use an open-source project such as Starburst and ITU Gaze Tracker (Ryan et al., 2008a; Villanueva et al., 2009; Deng et al., 2010; Tafaj et al., 2011) (for Starburst and Gaze-Tracker methods refer to Li et al. (2005) and San Agustin et al. (2010), respectively).

Two major features prevent these methods from being used in low-cost eye-tracking applications, especially for mobile applications: (1) Most of the methods achieving reasonable performance can only be used by researchers with access to the platform in which they were developed. By way of example, Starburst is provided only in MATLAB and Gaze-Tracker in C#. One can implement these methods in any platform as details of their approach is available in the literature (see Ryan et al., 2008b for C/C++ implementation of Starburst.), nevertheless, this assumes an appropriate level of programming expertise in the user. (2) Most pupil detection algorithms with high-precision suffer from trade-offs in speed or accuracy, e.g., Campadelli et al. (2006) presented a fairly accurate method of localization of eyes in which the speed of processing is sacrificed for accuracy (4 s per frame). Most of these methods are based on an iterative algorithm. Repetition of this algorithm increases processing time, leading to lower processing speeds. In contrast, we developed an algorithm that uses simple computational steps without iteration. This simplicity allowed us to be able to develop the method in multiple platforms for the ease of application of the method by researchers with different needs.

We have implemented an open-source, multi-platform method known as “SET” (Sinusoidal Eye-Tracker) that exhibits superior performance, in terms of both speed and accuracy, not just indoors, but even in challenging mobile, outdoor environments. The key feature of SET is the proper combination of manual and automatic steps that achieves high precision with a reasonable speed. Only two parameters are adjusted prior to pupil detection: (1) the threshold for conversion of the eye image to a black and white image and (2) the size of the segments considered for pupil detection. Additionally we used an approximate ellipse-fitting algorithm to detect ellipse-like contours (i.e., the pupil) in the binary image, thereby facilitating location of the center of the pupil. We compared SET with the two previously mentioned open-source methods using two different sets of images: one extracted from videos taken in outdoor environments; the other from a dataset of images taken in controlled laboratory settings (“CASIA-Iris-Thousand”).

## Methods

Our SET pupil detection method was originally implemented in MATLAB 2006a (MathWorks® Inc., www.mathworks.co.uk) and relies upon the Image Processing toolbox in MATLAB (www.mathworks.co.uk/products/image). From the many open-source methods available (wiki.cogain.info/index.php/eye_trackers), we selected Starburst and Gaze-Tracker to compare SET against, as these are the most commonly used.

Starburst is implemented in the openEyes open-source toolkit (thirtysixthspan.com/openeyes/videos.html), which was developed in MATLAB (Li, 2006). This method is designed for eye-tracking in a sequence of images. It uses the detected pupil position in the previous frame as the starting point of the search in the current frame. In cases where there is no prior pupil position, the center of the image is considered as the starting point. Starburst uses a ray casting method to find points on the border of the pupil (candidate feature points). Subsequently it uses these points to establish a feature point consensus set using random sample consensus (RANSAC). The best-fitting ellipse is determined using the consensus set. Finally a model-based optimization is applied to optimize the ellipse parameters. In order to achieve the best performance of this algorithm, we used the manually selected pupil-center point (“PCP”) as the beginning point (“Starburst Exact”). For a detailed explanation of this method with different inputs refer to Appendix 3 in the Supplementary Material.

Gaze-Tracker (www.sourceforge.net/projects/gazetrackinglib) was developed in C# (Visual Studio.net) and employs Open CV for image processing (San Agustin, 2009). This method is a complete system from image extraction to determination of the PoR. The first four steps include: (1) extraction of the face from the rest of the image, (2) detection of the eyes in the face, (3) detection of the PCP and finally (4) gaze estimation. To compare it directly to the pupil detection algorithm used in SET, we extracted the procedure for the detection of the PCP (step 3) out of Gaze-Tracker and fed the same eye images to this procedure as well as to SET. Similar to Starburst, Gaze-Tracker uses the RANSAC procedure to eliminate possible outliers amongst points in the contour between pupil and iris.

The processing was performed using a PC with Intel® Core 2 DuoTM 2.66Hz CPU, 3GB of RAM and Windows XP 32-bit operating system. This low specification computer was used for data analysis in order to investigate the efficiency of our algorithm.

### Image collection

Natural Frames. Videos were taken from 5 participants who were guided through an approximately 30 minute walk in London streets close to the UCL Institute of Cognitive Neuroscience. Lighting conditions therefore vary considerably between participants and even within one video, dependent on changes from cloud-cover continuously moving across the sun and participants walking from shaded to bright areas amongst other things. Participants wore a head-mounted eye-tracker manufactured by Positive Science Eyetracking (www.positivescience.com), which held an infra-red (“IR”) camera facing the right eye, an IR light emitting diode (“LED”) to illuminate the eye, and a digital handycam with 640 × 480 pixel resolution taking images of the scene observed by the participant at 30 frames per second.

These “Natural” frames were scaled to 640 × 480 pixels to achieve the original 4/3 width to height ratio as captured by the camera. A total of 74 frames were discarded due to low visibility of the pupil caused by blinks, saccades and images with massive distortion due to the high variation in lighting conditions in an external environment.

**Figure 1:**
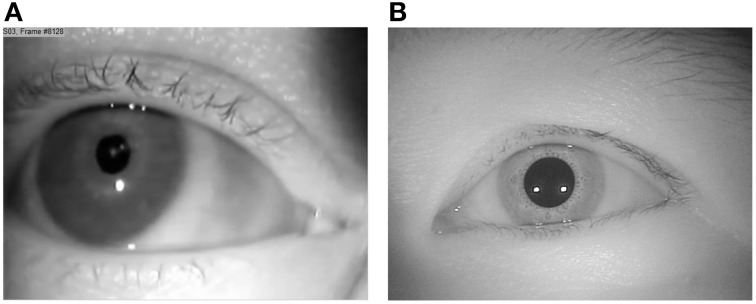
**Sample images of (A) our own (referred to as “Natural”) and (B) CASIA-Iris collection**.

CASIA-Iris Frames. We extracted 500 random frames from the CASIA-Iris-Thousand website (biometrics.idealtest.org) collected by the Chinese Academy of Sciences' Institute of Automation (CASIA). This website shares general biometric data in order to facilitate research and development on iris recognition and pupil detection methods. CASIA-Iris-Thousand includes frames of both left and right eyes of 1000 participants with more than 20,000 frames at a resolution of 640 × 480 pixels taken in laboratory settings. An example of frames from each of these collections is shown in Figure 1.

### Pupil detection

The SET pupil detection method consists of the following steps:

1. Thresholding.
2. Segmentation.
3. Border Extraction using Convex Hull method (Cormen et al., 2009).
4. Decomposition of each segment into sinusoidal components.
5. Selection of the segment with the best fit.

**Figure 2:**
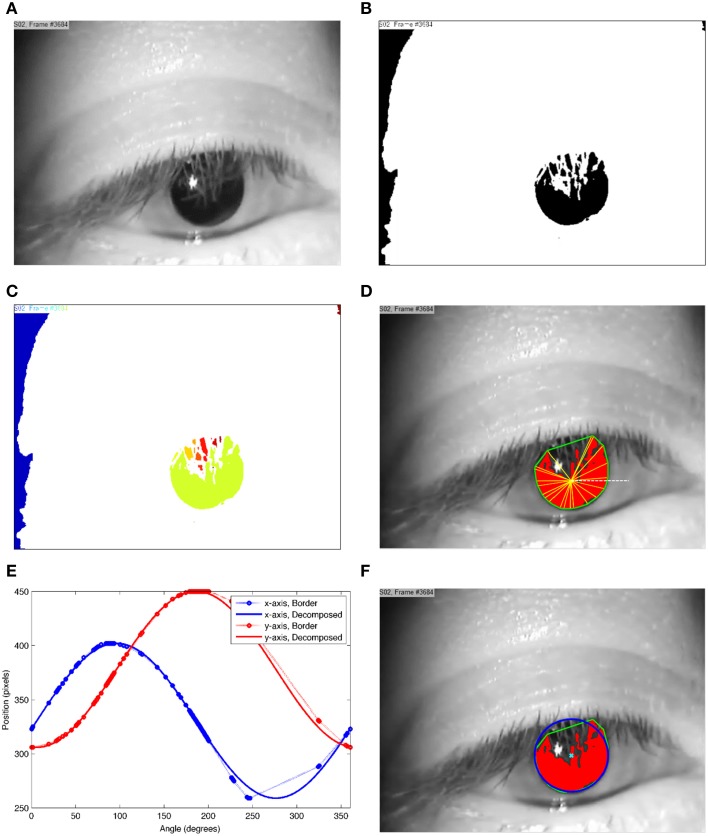
**Processing steps to extract the pupil center point. (A)** the original grayscale image; **(B)** the black and white thresholded image; **(C)** the segmented image in which each segment is displayed in a different color; **(D)** a highlighted segment (red) with its border extracted using Convex Hull method (green border), which actually covers some part of the pupil; The angle of each point on the border is calculated using the yellow lines and the horizontal white line that meet at the center of the segment **(E)** the decomposition of the shown border into its sinusoidal components with each point being an edge on the green border shown in **(D)**; and **(F)** the extracted pupil (blue ellipse) and the estimated pupil center point (cyan cross).

The first step is to extract the areas in which the pupil may be located by thresholding (Figure 2A). This step results in a binary image (black and white image) that most likely includes the pupil and possibly some other areas that could survive the threshold such as dark eyelashes and corners of the image (Figure 2B). The threshold can potentially take any value between zero (black) and 255 (white).

Using the “bwlabel” function in the MATLAB Image Processing Toolbox, we segmented the image into unconnected areas, Figure 2C. bwlabel returns a matrix the same size as the input black and white image, containing labels for the connected segments (Haralick and Shapiro, 1992, pp. 28–48). bwlabel uses connected components labeling of the binary image to group pixels into maximal connected regions. It gives a unique label to all pixels with binary value 1 that are connected to each other by a path of pixels.

The total number of pixels in one segment is considered to be the size of the segment. Subsequently the following steps are taken for every segment bigger than a certain value. The borders of the segments are extracted using the Convex Hull method, Figure 2D. MATLAB uses the Quickhull algorithm designed by Barber et al. (1996). The border of each segment is then used to estimate the matching ellipse [see equation (1) for calculation of *x*(ω) and *y*(ω)].

The points along the border are not uniformly distributed over a 360° circular angle due to discontinuities in the pupil image. Figure 2E shows an abrupt change in *x* and *y* values within the interval 200°−360°. To correct for this non-uniform distribution of values, we considered the center of the extracted border, the arithmetic mean of the *x* and *y* values, as a reference point and calculated the angle of each point in relation to the horizontal line. This angle is subsequently used to describe the distribution of points in the *x* and *y* axes along the border. Figure 2E shows how the points are distributed over a 360° circular angle. These angles and positions in the *x* and *y* axes are then used to estimate the covering sin and cos components.

An upward ellipse—an ellipse where the major axis aligns with vertical axis—can be defined as follows:

(1) $$\{\begin{array}{c} x(\omega )=h\;-\;b\sin(\omega )\\ y(\omega )=k\;+\;a\cos(\omega ) \end{array}$$

in which the pair (*h,k*) and values *a* and *b* represent the center of the ellipse, the semi-major and semi-minor axes respectively. For a detailed description of the definition of an ellipse refer to Appendix 1 in the Supplementary Material. We fit Equation 1 to the extracted points along the border to extract the free parameters ω, (*h,k*), *a* and *b*.

Using Taylor expansions we reduce the time to make each fit substantially, as calculation of polynomial expressions such as those in Equation (2) is much less computationally demanding than calculation of *sin* and *cos* functions. To reduce the complexity of estimation, we used only the first 3 terms (*n* = {0, 1, 2}) of the Taylor expansion of *sin* and *cos* components:

(2) $$\begin{array}{l} \sin(\omega )=\sum_{n=0}^{2}\frac{{\left(-1\right)}^{n}}{(2n+1)!}\omega^{2n+1}=\omega -\frac{\omega^{3}}{3!}+\frac{\omega^{5}}{5!}\\ \cos(\omega )=\sum_{n=0}^{2}\frac{{\left(-1\right)}^{n}}{(2n)!}\omega^{2n}=1-\frac{\omega^{2}}{2!}+\frac{\omega^{4}}{4!} \end{array}$$

The first 3 terms are the minimum number of terms that result in a reasonable accuracy. Higher numbers of terms were also tested but the results were not significantly different. Therefore, the fitting procedure reduces to fitting a polynomial to the data on each axis. Figure 2F shows the pupil (red segment) along with its covering ellipse (blue ellipse) and the calculated center (cyan cross).

One ellipse is fitted to each extracted segment (Figure 2E). Out of the entire calculated ellipses, the one with *a/b* being closer to unity, i.e., the ellipse that is closest to a circle, is considered as the pupil and (*h,k*) is reported as the center of the pupil (Figure 2F).

Two manually assigned values are used in our method: (1) the value for thresholding the image into a black and white image and (2) the value for inclusion of regions in further analysis. The former value depends on the overall luminance of the pupil—although the pupil is usually black, it is not necessarily black in the images. Therefore, a value higher than zero should be used to control for this. The other manually adjusted parameter depends on the overall size of the pupil in the images. This depends on the physical setup of the camera, the view angle and its distance to the eye. The threshold values of 30 and 600 respectively, were used in all the analyses reported in this paper.

### Detection of point of regard (PoR)

**Figure 3:**
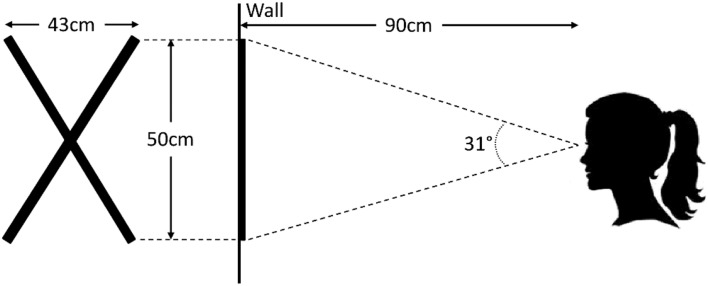
**Calibration cross (on the left) used for calibrating the eye and scene cameras to map pupil center point (PCP) to point of regard (PoR)**.

In order to be able to compare the SET detection method with other methods in which the accuracy is given in terms of visual degree of gaze, we used calibration data to transform PCP to point of regard (PoR) for videos recorded in outdoor environments (Zhu and Ji, 2007). Participants stood approximately 80–90 cm in front of a calibration cross that extended 69 cm along each line. Participants were asked to fixate at 5 points marked on the cross using a cue. Calibration was performed separately for each video. Figure 3 shows an image of the setup of the calibration. The calibration procedure was done using a graphical user interface (GUI) developed in MATLAB (Javadi et al., Unpublished). We should emphasize that this is a simplified method of detection of the PoR. For more elaborate methods, one should consider many other parameters such as glint and eye model (for a review see Morimoto and Mimica, 2005).

### Statistical analysis

Images in each collection (“Natural” and “CASIA-Iris”) were manually processed by two experimenters (AHJ and LT) independently, in order to detect the PCP in each frame using a graphical user interface (GUI) developed in MATLAB. The mid-point in-between their selected PCP for each image was used for further analysis, *p_m_* (*x_m_, y_m_*). *p_m_* (*x_m_, y_m_*) were considered as the ground truth and the performance of the algorithms were compared against these points. Detection error for each algorithm (“*e*”) was calculated by Euclidean distance between detected PCP, *p_d_* (*x_d_, y_d_*), and manually selected PCP:

(3) $$e=\left|\sqrt{{\left(x_{d}-x_{m}\right)}^{2}+{\left(y_{d}-y_{m}\right)}^{2}}\right|$$

Due to the asymmetric distribution of *e* (*e*≥ 0) and its concentration close to zero, we used a criterion based on exponential decay to label the frames as *hit* or *miss*. Exponential decay is described as follows

(4) $$f(x;p_{0},p_{s},p_{r})=\{\begin{array}{ll} p_{0}+p_{s}\exp(-p_{r}x) & x\geq 0\\ 0 & x<0 \end{array},$$

in which *p_r_* >0, *p_o_* and *p_s_* are rate, offset and scale parameters, respectively. For further discussion on this method refer to Appendix 2 in the Supplementary Material. We fitted an exponential decay to the data for each model and image collection. *e* values smaller than *x*-intercept, (*p_o_* + *p_s_*)/*p_r_ p_s_*, of the line describing the slope of the decay were marked as *hit*. The rest were marked as *miss*. Only images marked as hit were used for further analysis.

Processing time was also recorded for each method in their native programming environment, i.e. MATLAB for Starburst and C# for Gaze-Tracker. Although the detection errors are consistent across platforms, the processing speed is not. Therefore, to have a valid comparison between methods, we compared the speed of SET in MATLAB with Starburst and in C# with Graze-Tracker. Processing times (in milliseconds) further than 2 standard deviations from the mean for each method and image collection were excluded from the analysis.

Statistical data analysis was performed using SPSS (v17.0; LEAD Technologies Inc., Charlotte, NC, USA). Data was checked for normality of distribution using Kolmogorov-Smirnov test. The test of normality showed that the distribution of detection errors was not normal in any of the methods and image collections (in all the comparisons: *p* < 0.03). Detection error was therefore subjected to a Kruskal-Wallis test with three levels of method (SET/Starburst/Gaze-Tracker), split over image collection (Natural/CASIA-Iris). The dependent variable was the detection error (“*e*”) as calculated in Equation (3). Subsequently *post-hoc* two-independent-samples Mann-Whitney *U*-tests were used to compare accuracy between different methods. Bonferroni-adjusted alpha level of 0.016 (0.05/3) was used as the significance threshold. Furthermore, absolute value of Cliff's Delta effect size measure (δ) was reported (Cliff, 1993; Hess and Kromrey, 2004; Macbeth et al., 2011). This measure relies on the dominance concept rather than the mean as in conventional effect size measures such as Cohen's *d* and is more robust under skewed distributed data. Interpretation of its values are similar to Cohen's *d* with conventional effect sizes of small, medium or large.

Test of normality for processing times showed that they are distributed normally. A Two-Way ANOVA with method (SET/Starburst/Gaze-Tracker) and image collection (Natural/CASIA-Iris) as independent factors was conducted on processing time. Partial eta square (η*_p_*^2^) was reported as a measure of effect size. Subsequently, two-independent samples *t*-tests were used to compare the methods. Similar alpha level of Bonferroni correction 0.016 (0.05/3) was used as significance threshold.

## Results

9 (1.80%) and 72 (14.80%) out of 500 frames were discarded from CASIA-Iris (mainly due to glasses) and Natural image collections, respectively. For samples of excluded frames refer to Appendix 4 in the Supplementary Material. Manual detection of the PCPs was quite consistent between the two experiments [mean distance (SD) = 1.76 (0.12) pixels].

### Detection rate

Detected PCPs (using the three methods) were classified as hits and misses using exponential decay criterion. Table 1 shows detection rate for each method and image collection. Based on this table, Gaze-Tracker had the lowest detection rate and SET and Starburst had comparable detection rates. More importantly, it shows that our method performed fairly consistently between Natural and CASIA-Iris image collections as opposed to Starburst which performed better in the CASIA-Iris image collection.

**Table 1:** **Threshold (pixels) used for classification of frames into hit and miss using exponential decay criterion and detection rate (%) for different methods and image collections**.

	**Natural**		**CASIA-Iris**	
	**Threshold**	**Detection Rate (%)**	**Threshold**	**Detection Rate (%)**
SET	3.57	85.23	5.92	83.41
Starburst	8.84	79.15	9.95	93.68
Gaze-Tracker	15.17	28.48	7.41	32.75

### Detection error

Images marked as miss were excluded from the rest of the analysis. Detection Errors were calculated using Equation (3). Detection errors for the remaining images were subjected to a Kruskal-Wallis test. This test showed a highly significant main effect of method for both image collections (*p* < 0.001). *Post-hoc* comparisons showed a significant difference between SET and the other two methods in both Natural and CASIA-Iris images (*p* < 0.001). There was, however, no difference between Starburst and Gaze-Tracker methods. Table 2 shows the summary of the comparisons along with Cliff's Delta effect size measure (δ).

**Table 2:** **Summary of comparisons of detection errors (two-independent-samples Mann-Whitney *U*-tests) between different methods split over the two image collections along with Cliff's Delta effect size measure (δ)**.

**Comparison**	**Natural**		**CASIA-Iris**	
	***p***	***δ***	***P***	***δ***
SET and Starburst	<0.001	0.22	<0.001	0.64
SET and Gaze-Tracker	<0.001	0.37	<0.001	0.69
Starburst and Gaze-Tracker	=0.21	0.09	=0.14	0.11

**Figure 4:**
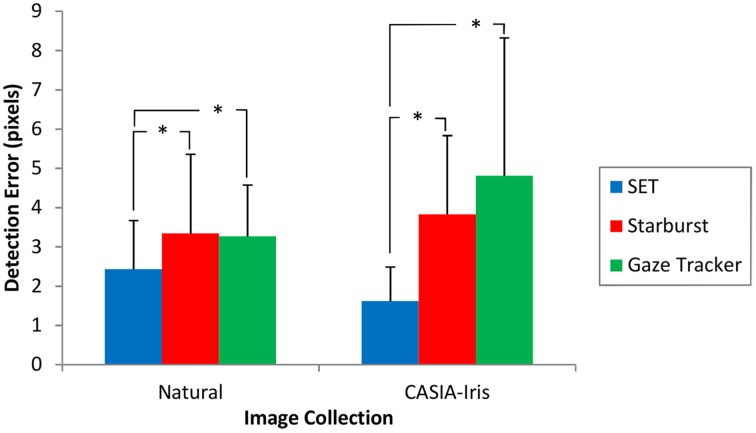
**Detection error for different methods and image collections after exclusion of missed frames**. Error bars reflect one standard deviation. ^*^p < 0.001.

**Figure 5:**
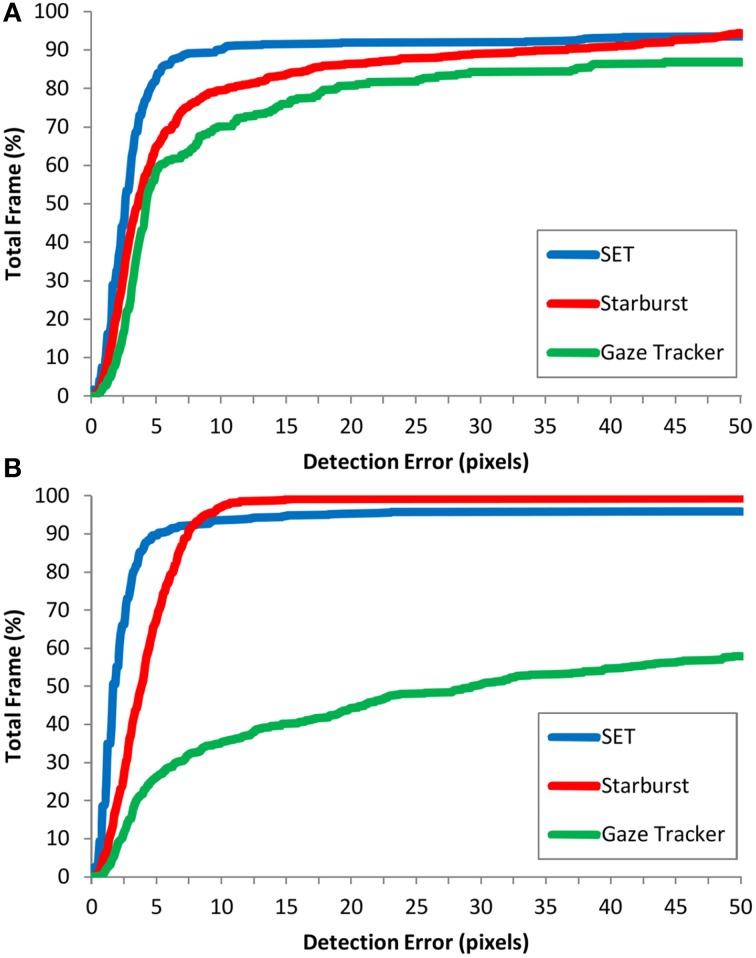
**The cumulative distribution of detection error for different methods for (A) Natural and (B) CASIA-Iris image collections**. y-axis shows the percentage of frames with detection error smaller or equal to a certain value in the x-axis.

Figure 4 shows the detection error for different methods and image collections. Figure 5 shows the cumulative distribution of detection error for different detection methods for different image collections. This figure shows that our method has the steepest initial slope. Additionally it shows that, although our method achieved higher initial slope, it was slightly outperformed by Starburst. It also shows a strong difference in performance of Gaze-Tracker for Natural and CASIA-Iris methods. For samples of different detection errors refer to Supplementary Material.

PCPs were mapped into PoRs for the three methods on Natural image collection. This mapping achieved a mean accuracy (SD) of 0.94° (0.47°) of visual angle for SET, 1.28° (0.77°) of visual angle for Starburst and 1.25 ° (0.50°) of visual angle for Gaze-Tracker.

### Detection speed

**Figure 6:**
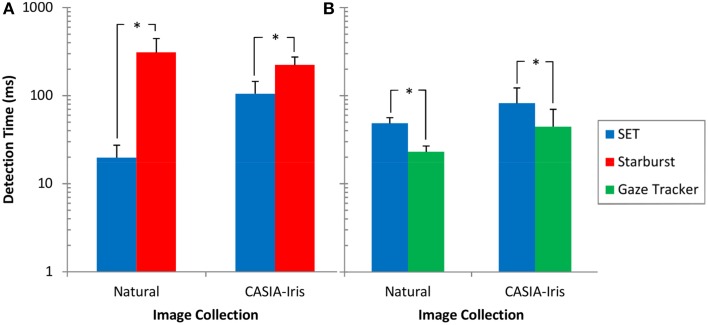
**Detection time for different methods and image collections. (A)** Comparison between SET and Starburst. For this comparison both SET and Starburst algorithms are run in MATLAB. **(B)** Comparison between SET and Gaze-Tracker. For this comparison both SET and Gaze-Tracker are run in C#. Error bars reflect one standard deviation. ^*^p < 0.001.

Data for frames with processing times longer than 2 standard deviations from the mean, was excluded from further analysis. Table 3 shows the percentage of exclusion of frames for each method and image collection. Non-parametric tests showed highly significant differences between the three methods. These comparisons showed that Starburst is worse than SET and SET is worse than Gaze-Tracker, Table 4. Figure 6 shows the detection time for each method split over each image collection.

**Table 3:** **Percentage of exclusion of frames based on excessive duration of processing time**.

	**Natural (%)**	**CASIA-Iris (%)**
SET	1.77	4.88
Starburst	4.46	1.42
Gaze-Tracker	0.51	1.79

**Table 4:** **Summary of comparisons of detection times (two-independent-samples Mann-Whitney *U*-tests) between different methods split over the two image collections along with Cliff's Delta effect size measure (δ)**.

**Comparison**	**Natural**		**CASIA-Iris**	
	***p***	***δ***	***p***	***δ***
SET and Starburst (MATLAB)	<0.001	0.99	<0.001	0.83
SET and Gaze-Tracker (C#)	<0.001	0.99	<0.001	0.99

## Discussion

Here we introduce a new pupil detection method (“SET”) based on the deconstruction of contours in black and white images into sinusoidal components. We applied our method and two other commonly used open-source methods, Starburst (Li et al., 2005) and Gaze-Tracker (San Agustin et al., 2010) to two collections of eye images extracted from videos taken from outdoor (“Natural” image collection) and laboratory settings (“CASIA-Iris” image collection), and compared the performance of the methods in terms of detection rate, accuracy and speed.

The SET detection rate was better than both Gaze-Tracker and Starburst for Natural image collections, making it a great choice for outdoor natural scene eye-tracking, see Table 1. Separately, the SET detection rate was better than Gaze-Tracker but worse than Starburst for the CASIA-Iris image collection, see Table 1. Nevertheless, SET still performs with a high rate of pupil detection (>80%).

In terms of accuracy, the experimental results showed that our method achieved better accuracy, i.e., lower detection error, than the other two methods, see Table 2 and Figure 4. Another strength of the SET method is that it is consistently applicable to both images acquired in laboratory settings and outdoor environments in terms of accuracy. Gaze-Tracker achieved a very small hit-rate and yet achieved a comparable accuracy to Starburst. It shows that this method achieves fairly good accuracy for a small proportion of the frames (classified as hits) and very bad accuracy for the majority of the frames (classified as misses). The key to SET's superior accuracy lies in the ellipse fitting mechanism: by constraining the algorithm to fit an ellipse, SET can infer the location of the pupil with only a few points to accurately locate the ellipse. Because of this, it is able to perform well under conditions such as changes in shadow (occlusion), small eyelid opening and odd camera angles.

In terms of speed, SET was worse than Gaze-Tracker, yet better than Starburst in both collections, Table 4. Nevertheless, the fractional loss in speed is far outweighed by the gain in accuracy and detection rate in comparison to Gaze-Tracker (see Table 1 and Figure 4).

Contrary to Starburst and Gaze-Tracker which are offered open-source in only one platform (Starburst in MATLAB and Gaze-Tracker in C#), SET can be integrated in MATLAB code as well as visual programming environments in Windows OS such as C# and visual Basic through dynamic-link library (DLL) (www.eyegoeyetracker.co.uk). DLLs provide a mechanism for shared code and data. They can be called within another code to run a function and return the values to the calling code. DLLs are independent of the programming language that they are developed in and the programming language that they are called from. Therefore, they provide an easy way of sharing code in between platforms and programming languages.

The first generation of mechanical eye-tracking systems (optical lever) were introduced five decades ago (Riggs et al., 1954; Riggs and Schick, 1968). Albeit recent progress in eye-trackers has led to ultra-high accuracy and ultra-high speed using lasers, this method is prohibitively expensive, cumbersome and can be invasive (Martinez-Conde et al., 2009; Sheehy et al., 2012), The current focus of the community for psychological research is non-invasive tracking of the eye using video taken from one or two eyes. Based on a review published by Hansen and Ji (2010), 68.92% of the eye-tracking methods use an image of the pupil to extract the direction of PoR. Many commercial solutions are available with different features and applications. As these systems are offered with high costs (mostly more than £35,000) attention toward low-cost solutions has been increased and many hardware and software solutions are proposed (Li et al., 2006; Schumann et al., 2008; Lemahieu and Wyns, 2011; Huang et al., 2013). In some cases the accuracy of a low-cost system is reasonable for many applications.

Most of the commercial eye-tracking solutions (combinations of hardware and software) achieve accuracy around 0.5 visual degrees. Major features of these systems, which are difficult to achieve using low-cost systems, are requirements of highly controlled environments such as a laboratory with controlled lighting conditions and the development of matching hardware and software. Almost all of the low-cost systems are based on cameras available cheaply in the market. Using these cameras the pupil is seen as a dark black patch. Usually some near-IR emitters have been used to improve the visibility of the acquired image. In most of the commercial systems, however, the pupil is seen as a very bright patch. This is achieved by placing some IR emitters very close to the axis of an IR-camera, leading to reflection of the IR light directly to the camera due to the photoreflective nature of the back of the eye. This enables those systems to detect the pupil much more easily as there are fewer bright spots in the image compared with dark spots as in custom built systems. There are also systems in which a combination of both bright- and dark-pupil techniques is used (Morimoto et al., 2002). This system, however, achieved an accuracy of around 2° visual angle.

A diverse range of approaches has been proposed, such as algorithms using support vector machines (Crisafulli et al., 2009) or neural networks (Torricelli et al., 2008). The accuracy of most of these algorithms, however, is still around 1° of visual angle (Canessa et al., 2012). We used a dark-pupil technique and achieved accuracy close to 1° visual angle. Based on Hansen and Ji (2010), the accuracy of our method is better than 62.96% of, and comparable with 25.93% of published studies.

Hansen and Ji (2010) showed that only 37.84% of approaches are suitable for images acquired in outdoor environments. Our experimental results showed that our method is effectively applicable to images acquired from outdoor environments. Detection rate (Table 1) and cumulative distribution of detection error (Figure 5A) clearly shows that SET is the superior method for processing of outdoor environments.

One of our primary goals was to develop a method that possesses reasonably fast processing speeds without sacrificing accuracy. Running SET in MATLAB achieved a frame rate of approximately 50.71 fps (frame per second) and 9.54 fps for processing of images from the Natural and CASIA-Iris image collections, respectively. Running SET in C# achieved a slower frame rate of proximately 20.61 fps and 12.20 fps for processing of images from the Natural and CASIA-Iris image collections, respectively. Gaze-Tracker, on the other hand, achieved 43.46 fps and 22.57 fps for each image collection, respectively. Starburst was extremely slow with 3.23 fps and 4.48 fps for each image collection, respectively. These results show that processing times for SET were faster than Starburst and slower than Gaze-Tracker for both environmental conditions. Considering the low Gaze-Tracker's detection rate of 28.48% and 32.75% for Natural and CASIA-Iris image collections, respectively, vs. 85.23% and 83.41% for SET, we argue that this method sacrifices accuracy to a high extent in order to achieve higher speed. SET is still much faster than Starburst and some other methods such as the one proposed by Deng et al. (2010).

SET is a pipeline and the efficiency of each stage of processing highly depends on the efficiency of the previous stages. We based our algorithm on this sequential method to circumvent avoidable repeated calculations, for the sake of speed without compromising on accuracy. Our algorithm considers only two constants: (1) threshold value and (2) minimum segment size. These are very low level parameters and their adjustment is effortless. Considering a couple of constants is not uncommon. For instance EyeLink (www.sr-research.com) also sets the dark/light threshold value at the beginning of tracking. Additionally we should emphasize that only one set of constants is used for the processing of images in both datasets throughout this study. SET's superior performance shows that the proposed algorithm is robust against variations such as pupil size and lighting without repeated adjustment of constant values.

In conclusion, SET is a novel method of pupil detection using images acquired from the eye extracted from readily available off-the-shelf cameras. SET was developed as a new standard in eye-tracking for variable lighting conditions as well as the new generation of low-cost and portable eye-tracking equipment. We applied SET to two image collections: Natural and CASIA-Iris, which are sets of images in outdoor and indoor environments and compared its performance with two other commonly used open-source projects (“Starburst” and “Gaze-Tracker”). Our experimental results showed that SET is more accurate than the other two methods with often higher detection rates. SET was slightly slower than Gaze-Tracker, although this is compensated for by its far superior accuracy. It is also faster than Starburst. SET was originally developed in MATLAB. For the ease of application it is recoded in C# and compiled into a dynamic-link library (“DLL”) in order to enable researchers who use Windows OS programming languages such as C# and Visual Basic to use the toolbox (www.eyegoeyetracker.co.uk).

## Author contributions

AJ and LT designed and implemented the algorithm in MATLAB; MB and ZH implemented the algorithm in C#; AJ, LT, MB, and ZH analyzed the data; AJ, LT, MB, VW, and ZH wrote the manuscript.

### Conflict of interest statement

The authors declare that the research was conducted in the absence of any commercial or financial relationships that could be construed as a potential conflict of interest.
