# Supplementary material for: SET: a pupil detection method using sinusoidal approximation
Source: Front Neuroeng. 2015 Apr 9;8:4. doi: 10.3389/fneng.2015.00004 (PMC4391030; doi:10.3389/fneng.2015.00004)
Supplement: Supplementary file 1 [file Presentation1.PDF]

Appendix 1

An ellipse can be fully described by the following parametric equation.

$$\begin{cases} x(\omega) = h + a\cos(\omega)\cos(\theta) - b\sin(\omega)\sin(\theta) \\ y(\omega) = k + a\cos(\omega)\sin(\theta) + b\sin(\omega)\cos(\theta) \end{cases} \quad \text{(App 1.1)}$$

in which the pair (h, k) represents the center of the ellipse, a and b represent the semi-major and semi-minor axes, respectively and θ represents the orientation of the ellipse as shown in Figure 1. Finally, $\omega = [-180..+180]$ covers the trigonometric circle.

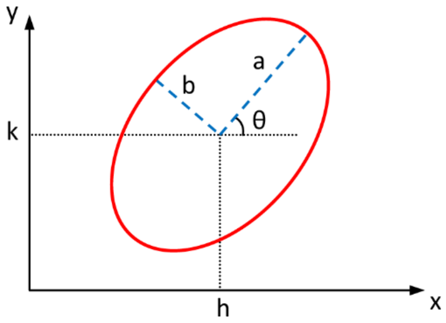

Figure 1. An ellipse and its constructing parameters.

Although x and y are composed of both *sin* and *cos* components, the overall shape of these two independent variables can be approximated fairly accurately by one component only. Decomposing x and y and plotting the components separately yields plots in Figure 2. Equations below show their decomposition.

$$\begin{cases} x_1(\omega) = a\cos(\omega)\cos(\theta) \\ x_2(\omega) = b\sin(\omega)\sin(\theta) \\ x_{comp.}(\omega) = h + x_1(\omega) - x_2(\omega) \end{cases} \text{, and} \quad \text{(App 1.2)}$$

$$\begin{cases} y_1(\omega) = a\cos(\omega)\sin(\theta) \\ y_2(\omega) = b\sin(\omega)\cos(\theta) \\ y_{comp.}(\omega) = k + y_1(\omega) + y_2(\omega) \end{cases} . \quad \text{(App 1.3)}$$

For the special case that $\theta = 90°$ the equations reduce to following equations

$$\begin{cases} x_1(\omega) = 0 \\ x_2(\omega) = b\sin(\omega) \\ x_{comp.}(\omega) = h - x_2(\omega) \end{cases} \quad \text{, and} \qquad \text{(App 1.4)}$$

$$\begin{cases} y_1(\omega) = a\cos(\omega) \\ y_2(\omega) = 0 \\ y_{comp.}(\omega) = k + y_1(\omega) \end{cases} \quad . \qquad \text{(App 1.5)}$$

Figure 3 shows the plots corresponding to $x_{comp.}$ and $y_{comp.}$ for the case $\theta = 90°$ and $h = 0$ and $k = 0$.

In order to reduce the complexity of the curve fitting we rotated the target area using a rotation matrix to match $\theta = 90°$. Having the eye-camera properly adjusted, however, the pupil is recorded very closely to a vertical ellipse. In this way a pair of components (x_1 and y_1) is enough to achieve a good estimate of the center of the matching ellipse.

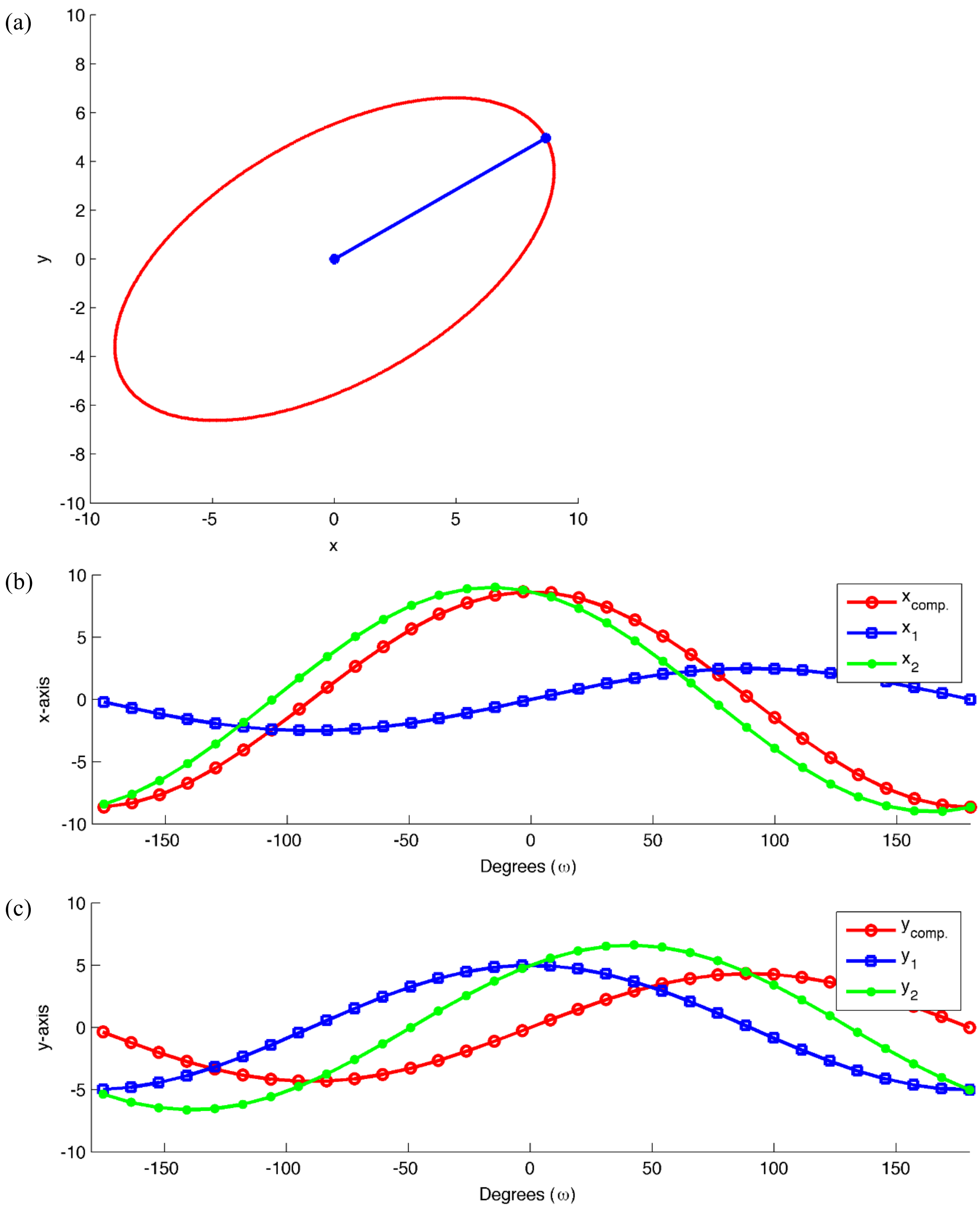

Figure 2. An ellipse (a) and its constructing components in x (b) and y (c) axes with $a = 10$, $b = 5$ and $\theta = 30°$.

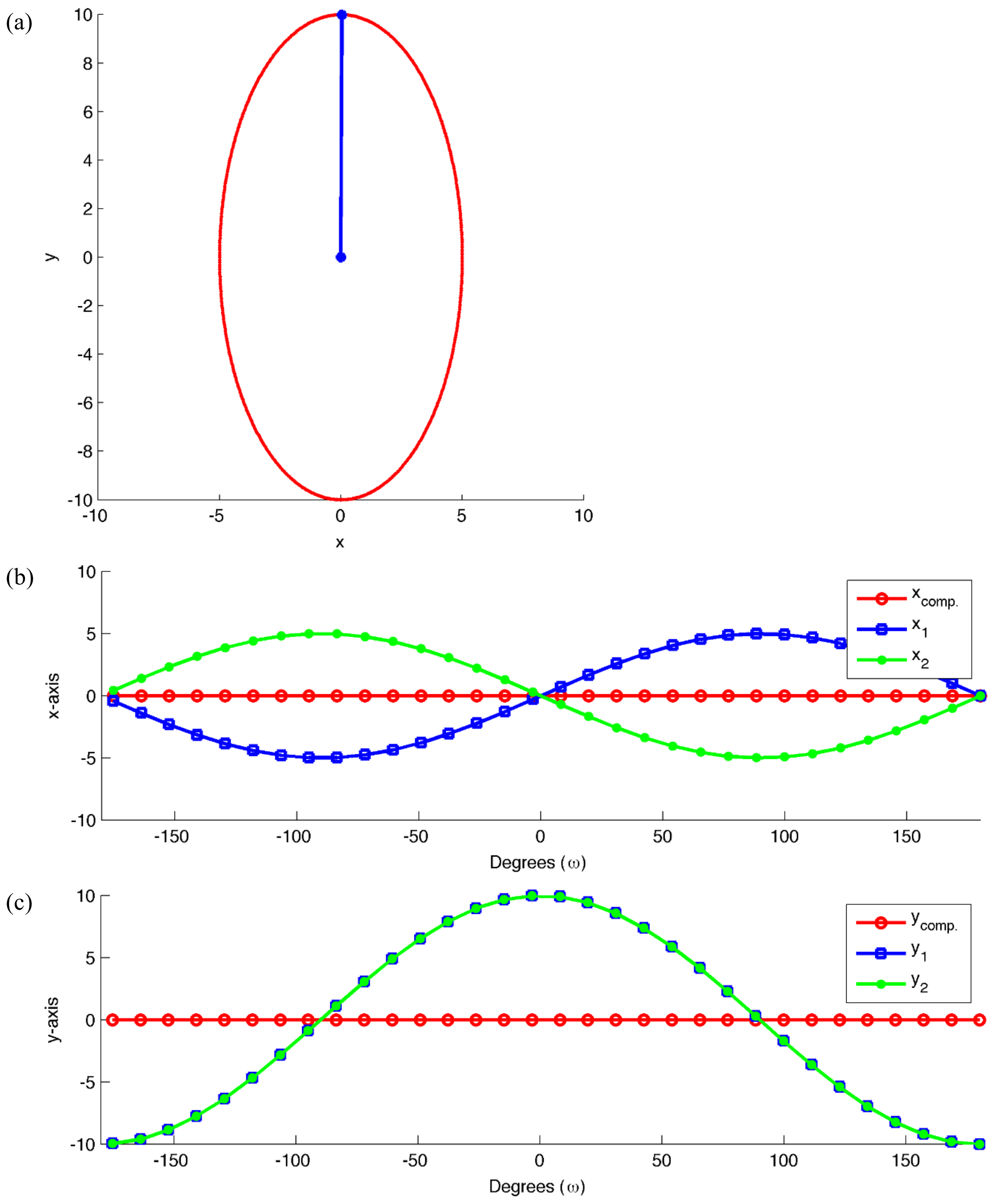

Figure 3. An ellipse in vertical orientation ($\theta = 90°$) and $a = 10$, $b = 5$.

Appendix 2

As mentioned, exponential decay, is described as follows

$$f(x; p_0, p_s, p_r) = \begin{cases} p_o + p_s \exp(-p_r x), & x \geq 0, \\ 0, & x < 0 \end{cases} \quad \text{(App 2.1)}$$

in which $p_r > 0$, p_o and p_s are rate, offset and scale parameters, respectively. The rate parameter ($\lambda = p_r$) in classical exponential decay, is equivalent to the inverse time constant ($1/\tau$), $\lambda = p_s = p_r$. Additionally it is assumed $p_o = 0$. Figure 4 shows the fitted exponential decay to the histogram of detection error ('e') for the Starburst method on the Natural image collection.

The x-intercept of the line describing the slope of the exponential decay can be calculated as follows. The slope of the exponential decay is defined as differential of $f(x)$ at $x = 0$.

$$d_f / d_x = (-p_r p_s) \exp(-p_r x) \quad \text{(App 2.2)}$$

$$d_f / d_x \big|_{x=0} = -p_r p_s \quad \text{(App 2.3)}$$

As $f(x)\big|_{x=0} = p_o + p_s$, the line describing the slope at $x = 0$ is as follows

$$y - (p_o + p_s) = (-p_r p_s)x. \quad \text{(App 2.4)}$$

Therefore with $y = 0$ the x-intercept (x_0) will be $(p_o + p_s) / p_r p_s$.

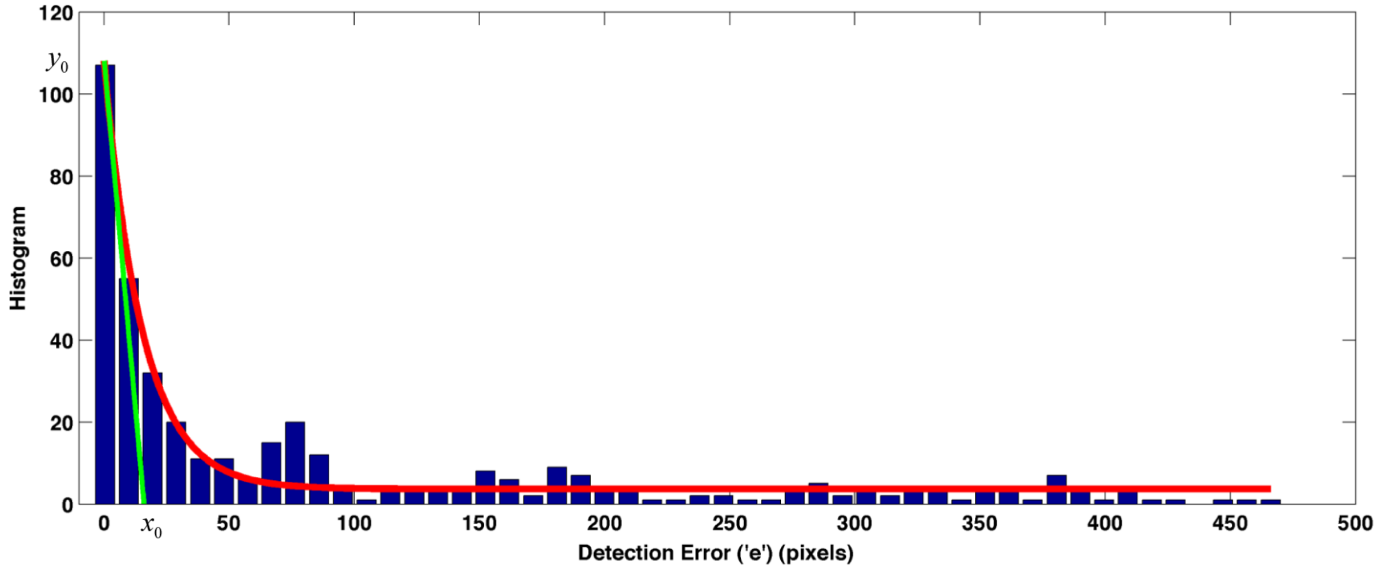

Figure 4. The histogram of detection error (‘e’) for Starburst method on Natural image collection. The red curve shows the fitted exponential decay and the green line (Eq. App 2.4) shows the slope at $x = 0$. This line is used to calculate the criterion for hits and misses. $x_0 = (p_o + p_s) / p_r p_s$ and $y_0 = f(x)|_{x=0} = p_o + p_s$.

Appendix 3

Starburst assumes that the PCP in the current frame must be close to its position in the previous frame. Therefore to reduce the search area, it considers the PCP in the previous frame as the starting point of its search method. It, however, resets the search point to the center of the image and continues the search from that point if the prior assumption does not lead to an acceptable solution. As we wanted to process frames separately, we ran Starburst for three different methods with different initial priors: (a) manually selected pupil center point (‘Exact’), (b) a point randomly selected around the manually selected pupil center point with a radius of 25 pixels (‘Random’) and (c) no prior which is equivalent to considering the center of the image as starting point (‘Center’). The first two methods achieved the best results. Figure 5 shows detection error (‘e’) for Starburst with different priors. As shown, the only difference lies in Starburst with no prior in detection of PCP in CASIA-Iris image collection. Figure 6 shows the distribution of detection error for different priors for different image collections. This figure shows that there is almost no difference in between Exact and Random priors.

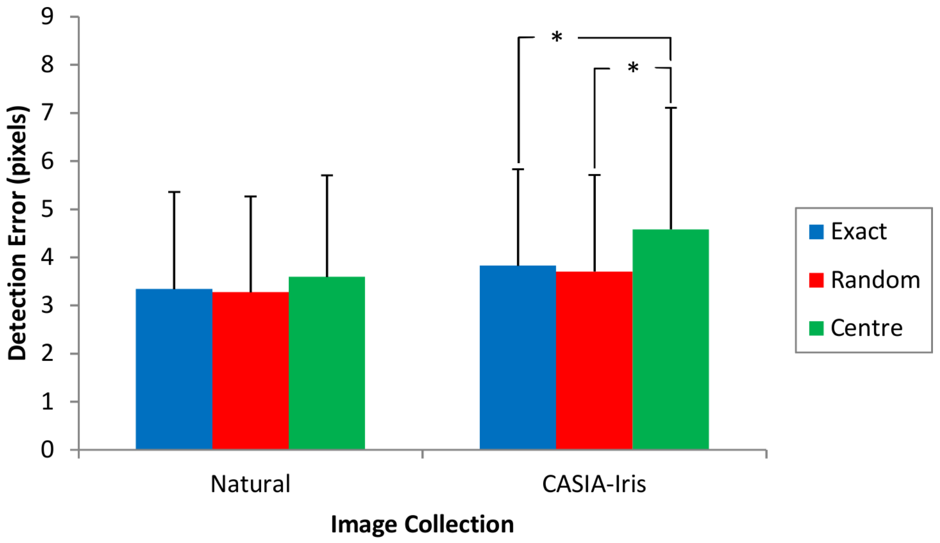

Figure 5. Detection error for Starburst algorithm with different priors and image collections after exclusion of missed frames. Error bars reflect one standard deviation. * p < 0.001.

(a)

(b)

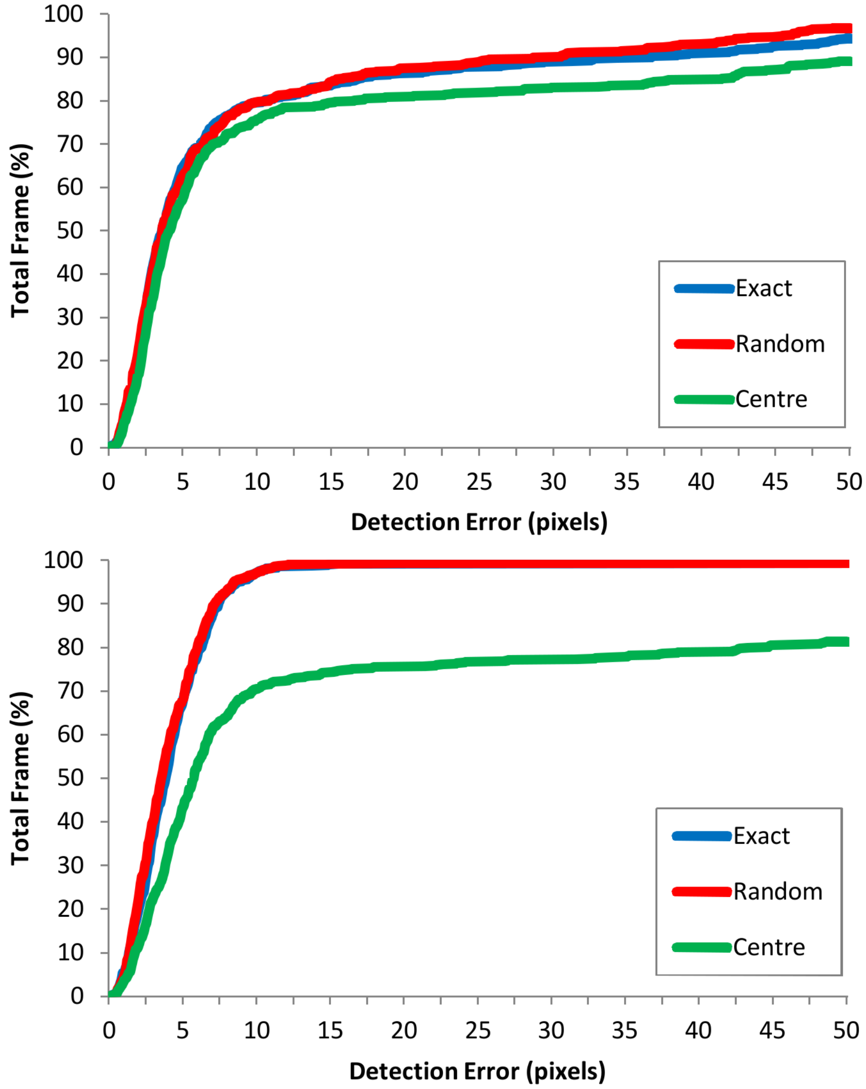

Figure 6. The cumulative distribution of detection error for Starburst algorithm with different priors for (a) Natural and (b) CASIA-Iris image collection.

Appendix 4

Samples of excluded images from the two image collections are shown in Figure 7. Figure 8 gives examples of cases that SET performed better and worse than other methods. In Figure 8a-b SET outperformed the other two methods. SET was not able to come up with any solution for Figure 8f.

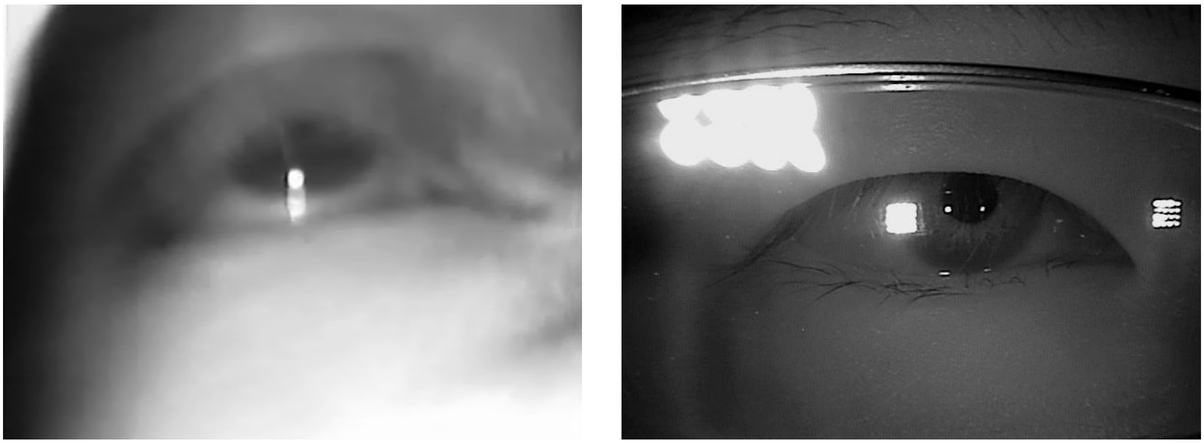

(a) (b)

Figure 7. Samples of excluded images from (a) Natural and (b) CASIA-Iris image collection due to blinking and excessive reflection, respectively.

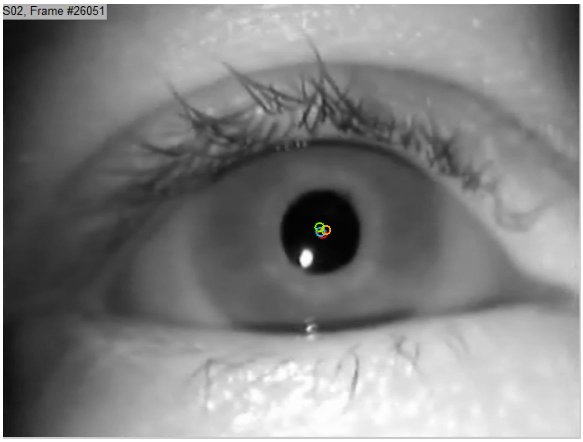

(a)

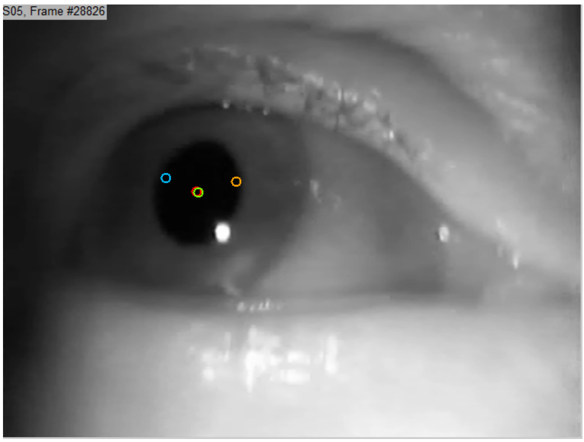

(b)

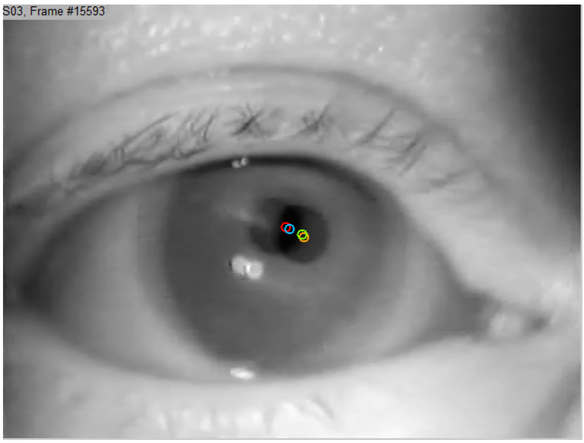

(c)

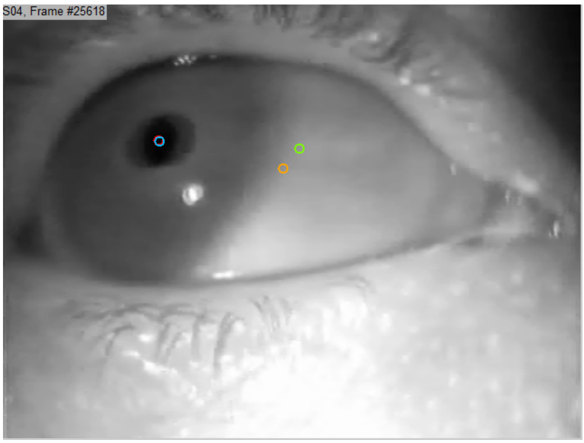

(d)

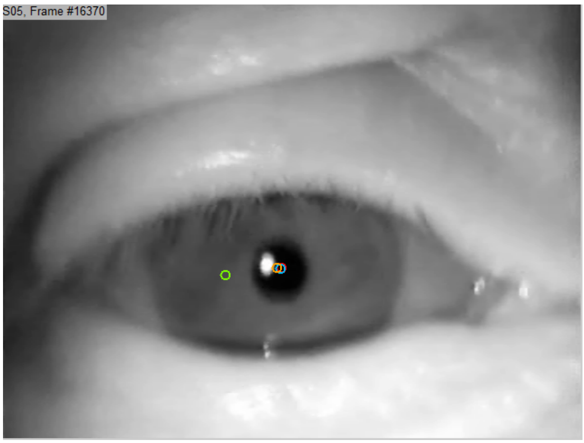

(e)

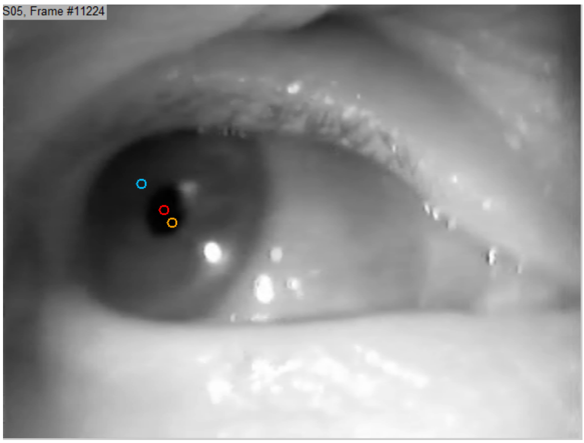

(f)

Figure 8. This figure compares the performance of the three methods in different conditions. Red, green, blue and brown circles represent location of manually selected pupil center point (PCP) and PCP indicated by SET, Starburst and Gaze-Tracker algorithms, respectively.
